# Supplementary material for: Patterns of Intron Gain and Loss in Fungi
Source: PLoS Biol. 2004 Nov 30;2(12):e422. doi: 10.1371/journal.pbio.0020422 (PMC532390; doi:10.1371/journal.pbio.0020422)
Supplement: Table S1 — Also available at http://genes.mit.edu/NielsenEtAl/. (4.3 MB ZIP). [file pbio.0020422.st001.zip › NielsenEtAl/html/207.html]

AN9425.1.NCU03086.1.MG01270.1.FG01221.1


```
 CLUSTAL W (1.82) Multiple Sequence Alignments - Introns Inserted


Sequence 1: NCU03086.1	276 aa
Sequence 2: MG01270.1	261 aa
Sequence 3: FG01221.1	285 aa
Sequence 4: AN9425.1	273 aa
Alignment Length: 307 aa
Number Identitical Residues: 63 aa
Alignment Score (without introns) 4271


MG01270.1 	------MSTMQQANRLKQAFMTK-KPAFGLWQM~LPGSNISRALARTP-----GIDWVMV
NCU03086.1	MSSGNPTSKMLAANSLKQIFTQESRPAMGVWQM~LPGANISRILARS------GADWVMV
FG01221.1 	------MSAMQQSNRLRTALLEG-KKAFGAWQM~IPGANVSRILARS------GVDWVLV
AN9425.1  	---------MALNTALNTALQTS-KTAYGFWLT2LPGAGVAKTILRSTAQHANRFSWVLV
          	         *   . *.  :    : * * *   :**:.::: : *:.:. :.  .**:*

MG01270.1 	DY1-------~AAMHEAVP---AIA~AVGVSPIVRIPDFQPWMVK-~--------~----
NCU03086.1	DC~EHGNIDD1AAMHEAVP---AIA~ALGVSPIVRIPDLQPSMVKR1ALDSGAH-~----
FG01221.1 	DC~EHGNIDD1GAMHDAVP---AIA~ALGVSPIVRLPDMQGWMVKR1ALDSGAHG0TDSD
AN9425.1  	DA~EHGLISD~KDYYEVDSYANPRS1SEGASPIIRVPWGEEWLIKR~ALDAGAHG~----
          	*  . .  ..    ::. . :.. : : *.***:*:*  :  ::*  : .:.: .     

MG01270.1 	-~--------L1QEVKTLVSACKFPPEGTRGFGSPFAMHTFSPTPSMTTYLQSANSSLLT
NCU03086.1	G0ILAPLIRTV~EDVKNLVTAAKFPPEGKRGFGSPIAMQNFNPMPTFTEYLQQANESLLT
FG01221.1 	K~IVVPLLRTP~EEARQLVQSAKFPPQGRRGFGSPIAPERFHPEPTFTQYLQQANNSLLT
AN9425.1  	-~ILTPMCHSE0EDAAKIVRYCKYPPQGSRGYGPMFALHSFPEVP-AAQYDAGADKNTAV
          	     .   :  ::.  :*  .*:**:* **:*. :* . *   *  : *   *:..  .

MG01270.1 	MVQIETREALERVAEIAPL--VDVVLVG~PFDLG~NNIGQPILDGVMSQELKDAVESVRV
NCU03086.1	MVQIETQEALDCVEEIAPL--VDVIFIG~PFDLG~NNIGHPIIEGKMDPELDEAIDRILK
FG01221.1 	IVQIETKEALESIDDIAAVNGIDVLFIG~PFDLG1NAIGHPIIEGVMASELKGAIAKILA
AN9425.1  	FVQIESKGGLESVEKIAAVEGLDGILIG1PFDLA~KQIG--VQRG--GDEHEAAIQRILK
          	:****:: .*: : .**.:..:* :::* ****. : **  :  *    * . *:  :  

MG01270.1 	AAERAGVVSGIFC~TGPEMAKSCADAGWGMISVATDLTALMGTMEERVAVARGEA1RFVQ
NCU03086.1	AAVTAGKKCGIFC~TGGEQAKQYADKGFHMISVATDYTLLGAAMADSLSIARGQG~---K
FG01221.1 	ASQKAGKKTGVYC~TGGEQAKVYADMGFDMMNVVTDYTSLALVAKEQLSFADGSS~APAR
AN9425.1  	AAKSARKKAAIFC1TDGADAQNRAQQGFDMVSIVTDVGVLGSGMLRELNVASGKA~E--G
          	*:  *    .::* *.   *:  *: *: *:.:.**   *       : .* *..     

MG01270.1 	A~EFLQTFLLFWIRLWYFNR
NCU03086.1	Q~DKPASY------------
FG01221.1 	G1FSSGAVDLLMCI------
AN9425.1  	G~KPRDGY------------
          	
```
